# A Novel Camel Milk-Derived Peptide LLPK Improves Glucose-Lipid Metabolism in *db*/*db* Mice via PPAR Signaling Pathway

**DOI:** 10.3390/nu17101693

**Published:** 2025-05-16

**Authors:** Binsong Han, Yuhui Ye, Cunzheng Zhang, Lina Zhang, Peng Zhou

**Affiliations:** 1 Jiangsu Key Laboratory for Food Quality and Safety-State Key Laboratory Cultivation Base of Ministry of Science and Technology, Institute of Food Safety and Nutrition, Jiangsu Academy of Agricultural Sciences, Nanjing 210014, China; hanbinsong@live.com (B.H.); yyh@jaas.ac.cn (Y.Y.); 2 School of Food Science and Technology, Jiangnan University, Wuxi 214122, China; zhoupeng@jiangnan.edu.cn; 3 International Joint Research Laboratory for Dairy Science and Technology, Jiangnan University, Wuxi 214122, China

**Keywords:** camel milk-derived peptide, diabetes, hepatic proteome, PPAR signaling pathway

## Abstract

**Background**: Camel milk is considered to be an important source of bioactive peptides with potential anti-diabetic effects. However, the mechanism by which these active peptides exert their anti-diabetic effects is not clear. The aim of this study was to systematically evaluate the in vivo anti-diabetic effects of Leucine-Leucine-Proline-Lysine (LLPK), a novel dipeptidyl peptidase-4 (DPP-4) inhibitory peptide identified from the in vitro gastrointestinal digestion product of camel milk. **Methods**: A *db*/*db* diabetic mouse model was used, and LLPK was administered to mice at doses of 50 mg/kg BW and 100 mg/kg BW as a daily oral gavage for 30 days. The effects of LLPK on fasting blood glucose (FBG), oral glucose tolerance test (OGTT), insulin tolerance test (ITT), and serum lipid levels were monitored, and possible mechanisms of action were elucidated using proteomics. **Results**: The results demonstrated that LLPK significantly improved diabetic symptoms, including FBG, OGTT, ITT, and serum lipid levels in *db*/*db* diabetic mice. Furthermore, significantly increased levels of serum glucagon-like peptide 1 (GLP-1) and reduced serum DPP-4 activity were observed in the LLPK-treated group compared to the control group. Hepatic proteomics indicated that LLPK improved glucose and lipid metabolism via the PPAR signaling pathway, where the key targets were Scd1, Acox1, Acaa1b, Slc27a1, Acsl1, and Ehhadh. **Conclusions**: In summary, this study provided new insights into the anti-diabetic mechanisms of camel milk and supported the development of camel milk-based anti-diabetic functional foods or nutraceuticals.

## 1. Introduction

Diabetes has become one of the most prevalent diseases in the world. Recent data indicated that the global number of adults with diabetes was 537 million in 2021, and was expected to be 783 million by 2045, posing a very serious risk to human health [1]. Of these, type 2 diabetes (T2DM), characterized by hyperglycemia and relative insulin deficiency, accounted for 90% of the total number of diabetes patients [2]. The current treatments for diabetes included insulin injection and various types of oral medications, e.g., sulfonylureas and biguanides. However, a certain amount of patients may suffer from side effects such as gastrointestinal upset and liver damage after long-term use of these medications [3]. Therefore, it is essential to develop safe, effective, and non-toxic natural products as alternative treatments for diabetic patients.

Bioactive peptides derived from dietary proteins are of great benefit to human health, particularly regarding diabetes, which has been confirmed by previous studies [4]. Milk proteins are considered one of the most important sources of bioactive peptides with potential anti-diabetic effects [5]. Further, camel milk has been recognized as a functional supplement due to its preventive and curative effects on diabetes [6]. Our previous study found that camel milk administered by gavage for one month to HDF/STZ-induced diabetic rats was effective in ameliorating lipid accumulation in the liver and lowering fasting blood glucose levels, and we further found that the ameliorative effect of camel milk in diabetic rats may be related to the activation of the hepatic AMPK signaling pathway [7]. A recent study also indicated that hydrolyzed peptides from camel milk could improve hyperglycemia by modulating the gut microbiota in diabetic mice [8]. Further evidence demonstrated that the hypoglycemic effects of camel milk may be linked to the DPP-4 and α-amylase enzymatic activities, which directly influence the hypoglycemic activity of peptides hydrolyzed from camel milk proteins. In addition, studies have been carried out to successfully identify DPP-4 activity inhibitory peptides from camel milk protein, indicating that camel milk-derived peptides exhibited high application prospect in treating diabetes [9,10]. In our previous study, hydrolyzed peptide fractions with the ability to inhibit DPP-4 activity were isolated from the in vitro simulated gastrointestinal digestion products of camel milk by molecular weight screening and HPLC separation. Then, 11 peptides were identified from them by UPLC-MS, among which, LLPK was the peptide with the strongest ability to inhibit DPP-4 activity (IC50 = 0.11 ± 0.01 mM, Supporting Information Table S1). These results showed that camel milk may be a new adjunctive therapeutic strategy for diabetes.

Although numerous studies have identified peptides from camel milk hydrolysate products that inhibit DPP-4 activity, most studies relied on in vitro enzyme viability assays or cellular assays. The in vivo efficacy regarding the potential hypoglycemic mechanisms in model animals have not been clearly elucidated. Therefore, the aim of the present study was to systematically evaluate the effects of LLPK on abnormalities of glucose-lipid metabolism in vivo in *db*/*db* diabetic mice. Our results demonstrated that LLPK inhibited serum DPP-4 activity and increased serum GLP-1 levels, leading to the improved FBG levels, OGTT, ITT, and serum lipid levels in *db*/*db* mice. Moreover, the hepatic proteome analysis indicated that the LLPK exerted protective effect on lipid metabolism via the regulation of the PPAR signaling pathway, thereby effectively reducing lipid accumulation in the livers of *db*/*db* mice.

## 2. Materials and Methods

### 2.1. Reagents

LLPK (≥98%) was synthesized by Nanjing Peptide Valley Biotechnology Co., Ltd. (Nanjing, China) using solid phase peptide synthesis. Acetonitrile (MS grade), formic acid (MS grade), and DPP-4 Activity Assay Kit (MAK088) were purchased from Sigma-Aldrich (City of Saint Louis, MO, USA). Other analytical grade reagents used in this work were all supplied by Aladdin (Shanghai, China). The fasting serum insulin (FIN), GLP-1 ELISA kits, and BCA protein assay kit were provided by Thermo Scientific (Waltham, MA, USA).

### 2.2. Animals

Male *db*/*db* mice (based on a C57BLKS/J background, *n* = 24) and non-diabetic male C57BLKS/J mice (*n* = 6) were obtained from SPF biotechnology Co., Ltd. (Beijing, China). During the animal experiment, all mice were kept in a specific-pathogen-free environment with a twelve-hour black/light cycle (room temperature: 25 °C). All mice were allowed to eat and drink freely.

### 2.3. Experimental Design

After one week of acclimatization, all normal C57BLKS/J mice were designated as the normal control group (NCG, *n* = 6), without any treatment. The *db*/*db* mice were divided into four groups randomly (*n* = 6/group): (a) diabetic control group (DCG), without any treatment; (b) metformin control group (PCG), gavage daily with 100 mg/kg body weight (BW) of metformin; (c) low-dose peptide treatment group (LPG), gavage daily with 50 mg/kg BW of LLPK; and (d) high-dose peptide treatment group (HPG), gavage daily with 100 mg/kg BW of LLPK. The mice in NCG and DCG were administrated the same volume of saline as the other groups. The animal experiment lasted for a total of 30 days, and body weight and FBG were measured every two weeks. OGTT and ITT were conducted on days 28 and 30, according to previous methods [11]. On the thirtieth day, all mice were sacrificed by dislocation after respiratory anesthesia using 5% isoflurane. Serum and tissue were then collected and stored at −80 °C for subsequent analysis. The Animal Experiment Ethics Committee of Jiangnan University approved this animal experiments (JN. No. 20221130c0880108[469]) on 16 December 2022.

### 2.4. Biochemical Assays

The serum levels of TC, TG, LDL-C, and HDL-C were determined using specific kits (obtained from Nanjing Jiancheng Bioengineering Institute, Nanjing, China). Additionally, serum levels of FIN, DPP-4 activity, and GLP-1 were assessed and calculated according to standard methods provided by the manufacturers’ instructions. The homeostasis model assessment index of insulin sensitivity (HOMA-IS) and insulin resistance (HOMA-IR) [12] were evaluated using the following equations:

(1) $$\mathrm{HOMA}-\mathrm{IR}=\frac{\mathrm{FIN}\;(\mathrm{mIU}/L)\times \mathrm{FBG}\;(\mathrm{mmol}/L)}{22.5}$$

(2) $$\mathrm{HOMA}-\mathrm{IS}=\frac{1}{\mathrm{FIN}\;(\mathrm{mIU}/L)\times \mathrm{FBG}\;(\mathrm{mmol}/L)}$$

### 2.5. Liver Histological Analysis

The histological analysis of liver tissue was based on our previous study [7]. At first, liver tissues were fixed in a 4% paraformaldehyde buffer, embedded in paraffin wax, and stained with hematoxylin-eosin according to the procedure. Finally, stained tissues were collected and photographed via light microscopy (Leica, Wetzlar, Germany).

### 2.6. Liver Protein Preparation and Digestion

Liver samples collected from the NCG, DCG, and HPG were divided into three biological replicates and subjected to protein preparation and digestion as previously described, with some modifications [13]. Specifically, the liver tissue was thoroughly mixed with urea (8 M), sodium deoxycholate (1%), and a protease inhibitor cocktail. The samples were then grinded three times for 30 s each using a high-throughput tissue grinder (Ningbo Scientz Biotechnology Co., Ltd., Ningbo, China). Subsequently, the grinded liver samples were centrifuged for 20 min under 4 °C and 16,000× *g*. After that, the supernatant was collected as protein samples for subsequent digestion. A protein sample of 100 μg was first reduced with 2 μL of TCEP (0.5 M), then alkylated with 4 μL of iodoacetamide (1 M), and finally digested overnight under 37 °C by trypsin with a ratio of 1/50 (enzyme/protein, mass ratio). After desalting on a C18 ZipTip column (Sigma-Aldrich, City of Saint Louis, MO, USA), the sample was lyophilized and stored until further analysis.

### 2.7. Nano-UPLC MS/MS Analysis

The total peptides in the digests of tissue proteins were analyzed by an UltiMate 3000 UPLC system (Thermo Fisher Scientific, USA) coupled with a tims-TOF Pro 2 MS (Bruker Daltonics, Bremen, Germany). A total of 200 nanograms of peptide samples was applied to a C18 column (15 cm × 75 μm i.d., 1.7 μm particle size, 120 Å pore size, IonOpticks, Notting Hill, Australia) and separated at a flow rate of 400 nL/min with the column temperature maintained at 50 °C. The mobile phases consisted of 0.1% aqueous formic acid solution (Phase A) and acetonitrile (Phase B). The detailed elution gradients were as follows: 0–15 min, 4–28% B; 15–19 min, 28–44% B; 19–23 min, 44–90% B; 23–26 min, 90% B; 26–30 min, 4% B.

In the parameter settings of the mass spectrometer, the data acquisition mode was set to DIA mode with an MS scan range of 350–1300 *m*/*z*, and isolation window width set as 40 Da. Furthermore, all precursor ions were fragmented in the collision cell at an energy of 40 eV for MS/MS data acquisition. Resolution was 120,000 in MS mode and 30,000 in MS/MS modes with AGC targets of 4 × 10^5^ and 2 × 10^5^, respectively.

### 2.8. Data and Statistical Analysis

Spectronaut 18 (Biognosys AG, Schlieren, Switzerland) software was applied to process the raw spectrum data from LC-MS/MS analysis. Specifically, trypsin was set up as the digestive enzyme and uniprot-mus_musculus (version 2022, 21,992 entries) was used as the sequence database. The Qvalue cutoff was set at 1% at the precursor level and protein level. The major groups abundance of peptides truncated by 1% Qvalue cutoff was then calculated using the MaxLFQ method. ANOVA analysis was introduced by SPSS 22.0 (BIM, Austin, TX, USA) to compare differences between groups, with a significant difference between groups indicated when *p* < 0.05. Bioinformatic analysis of significantly different expressed proteins (DEPs) was performed using DAVID.

## 3. Results

### 3.1. The Camel Milk-Derived Peptide LLPK Effectively Ameliorated Diabetic Symptoms in db/db Mice

**Figure 1:**
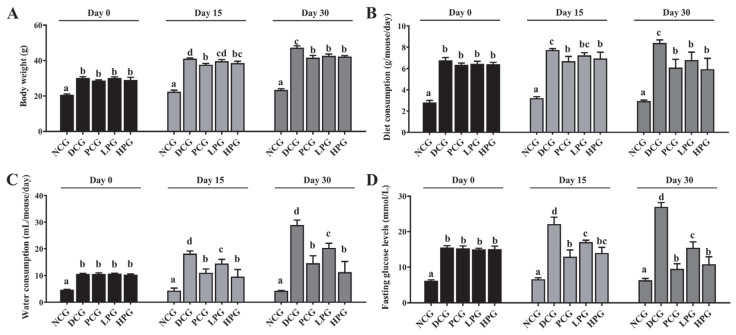
Effect of LLPK treatment on (**A**) body weight, (**B**) diet consumption, (**C**) water consumption, and (**D**) FBG in *db*/*db* mice (the letter superscripts indicate the significant differences among samples based on ANOVA analysis, *p* < 0.05).

As illustrated in Figure 1, *db*/*db* mice showed obvious diabetic symptoms. Specifically, *db*/*db* mice consumed more food and water than NCG mice, and at the same time, their body weight and FBG were significantly higher than those of NCG mice. However, after 30 consecutive days of LLPK treatment, the weight gain of LPG and HPG mice was significantly suppressed compared to DCG mice (Figure 1A, *p* < 0.05), and their food and water intake was significantly reduced (Figure 1B,C, *p* < 0.05). Meanwhile, LLPK treatment also significantly reduced FBG in diabetic mice. As shown in Figure 1D, FBG in LPG and HPG mice was significantly reduced by 42.71% and 59.96%, respectively, compared to that in DCG mice (*p* < 0.05).

**Figure 2:**
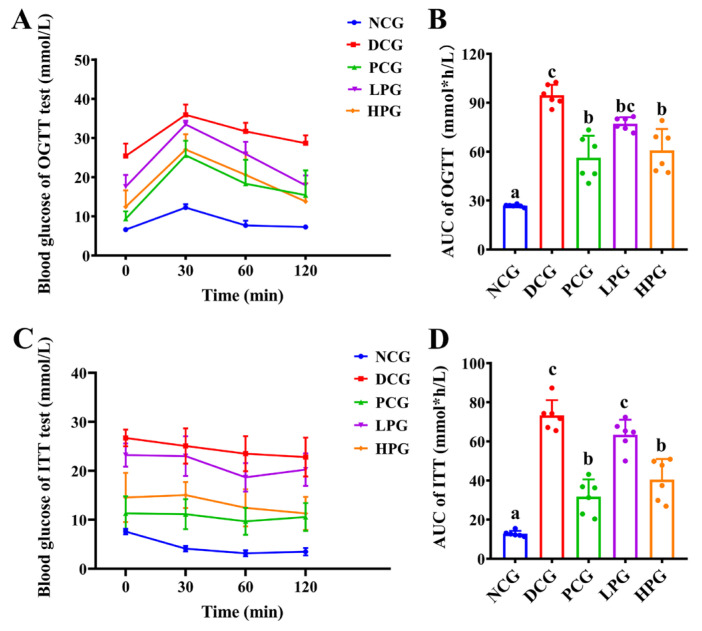
Effect of LLPK treatment on the glycemic homeostasis of *db*/*db* mice. (**A**) OGTT curves, (**B**) AUCs of OGTT, (**C**) ITT curves, and (**D**) AUCs of ITT (the letter superscripts indicate the significant differences among samples based on ANOVA analysis, *p* < 0.05).

The OGTT and ITT results further demonstrated the modulatory effect of LLPK on glycemic homeostasis of *db*/*db* mice. The results presented in Figure 2A,B indicate that the area under the curve (AUC) of the OGTT in DCG was significantly larger than that observed in NCG (*p* < 0.05), whereas it was significantly lower in the high-dose peptide treatment group (*p* < 0.05). In contrast, there was no significant difference between the LPG and the DCG. Similarly, the results from ITT (Figure 2D) indicated that the AUC for DCG was significantly greater than that of NCG (*p* < 0.05). Additionally, the AUCs for PCG and HPG were significantly lower than that of DCG (Figure 2D, *p* < 0.05), whereas no significant difference was observed in the AUCs between LPG and DCG.

It is well known that IR is a prevalent symptom of diabetes. HOMA-IR and HOMA-IS are widely used as indicators to assess the degree of IR. Table 1 demonstrated that HOMA-IR in DCG mice was significantly increased (4.77-fold, *p* < 0.05) compared to NCG mice, while HOMA-IS exhibited a significant decrease (*p* < 0.05) of 5.05-fold. These findings indicated that *db*/*db* diabetic mice have severe insulin resistance. Importantly, the HOMA-IR and HOMA-IS scores in PCG, LPG, and HPG showed significant improvement compared to DCG, with the improvement of HOMA-IR score in HPG being particularly higher than that of LPG (Table 1, *p* < 0.05).

### 3.2. LLPK Improved Serum Lipid-Related Indices and DPP-4 Enzyme Activity in db/db Mice

The development of diabetes is frequently accompanied by lipid metabolic disorders. Table 1 summarized the relevant indicators in mice serum and demonstrated that the serum TC, TG, and LDL-C levels in DCG were significantly raised (*p* < 0.05) when compared with NCG, while the serum HDL-C levels were significantly reduced (*p* < 0.05). These results indicated that DCG mice had obvious dyslipidemia. In contrast, after 30 days of continuous feeding, administration of LLPK significantly depressed the serum TC, TG, and LDL-C levels, while increasing HDL-C level. Among them, the ameliorative effect of high-dose LLPK intervention on TC, HDL-C, and LDL-C was more significant (*p* < 0.05) than that of low-dose LLPK. As shown in Table 1, serum DPP-4 enzyme activity increased significantly by 5.24-fold (*p* < 0.05) in DCG compared to NCG. Notably, after LLPK intervention, serum DPP-4 enzyme activity decreased significantly to 23.33 ± 1.82 (LPG) and 13.51 ± 0.33 (HPG) mU/mL, respectively. Correspondingly, the content of GLP-1 was significantly increased in HPG compared to DCG (*p* < 0.05).

### 3.3. LLPK Consumption Ameliorated Liver Damage in db/db Mice

**Figure 3:**
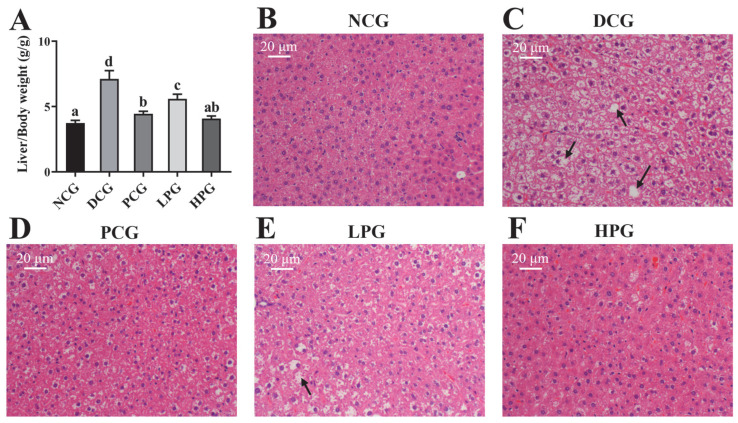
Effect of LLPK treatment on (**A**) liver/body weight and (**B**–**F**) liver histopathological injury of *db*/*db* mice (the letter superscripts indicate the significant differences among samples based on ANOVA analysis, *p* < 0.05, and the black arrows represent fatty degeneration).

The degree of liver hypertrophy was assessed by liver weight and the histopathological analysis of liver tissue. As illustrated in Figure 3A, the liver weights of DCG exhibited a significant increase compared to those of NCG. However, this increase was markedly reduced following the treatment with varying doses of LLPK, with the improvement being more pronounced in HPG compared to LPG. Furthermore, as shown in Figure 3B–F, liver tissues from NCG displayed no significant pathological changes, characterized by well-defined cell structure boundaries, normal morphology, uniform size, and well-defined nuclei. In contrast, liver cells from DCG exhibited abnormalities such as swelling, necrosis, and infiltration, as well as irregular cell shapes with a large number of fat vacuoles. Notably, after 30 days of intervention, liver cells from both LPG and HPG demonstrated normal morphology and structure, with improved vacuolization and aggregation of lipid droplets, and the tissue status of HPG was found to be superior than that of LPG.

### 3.4. LLPK Altered Liver Proteome Profiles in db/db Mice

**Figure 4:**
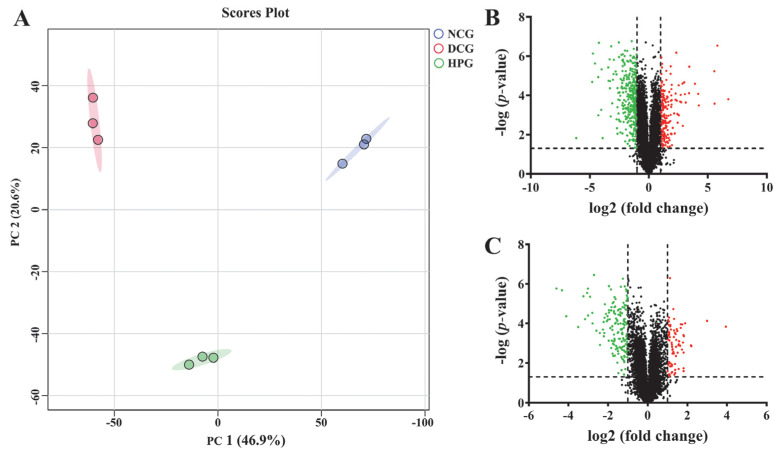
The effect of LLPK treatment on the hepatic proteome in *db*/*db* mice. (**A**) PCA analysis based on proteomics, (**B**) volcano plot between the NCG and DCG, and (**C**) volcano plot between the HPG and DCG (The green and red dots indicate down-regulated expression and up-regulated expression, respectively).

In this work, 6573 proteins were identified (Supporting Information, Table S2) through the DIA proteomics approach, and the data were quantitatively analyzed. As shown in the principal component analysis (PCA) results (Figure 4A), NCG, DCG, and HPG were clearly separated from each group, and the heterogeneity within each group was relatively low. Subsequently, we used a *p*-value < 0.05 and a fold change > 2 as the conditions for screening DEPs, and eventually 553 proteins (202 increased, 351 decreased, Figure 4B) were screened as DEPs between NCG and DCG, and 237 DEPs (75 increased, 162 decreased, Figure 4C) were identified between HPG and DCG.

### 3.5. LLPK Treatment Improves Lipid Metabolism in db/db Mice via PPAR Signaling Pathway

**Figure 5:**
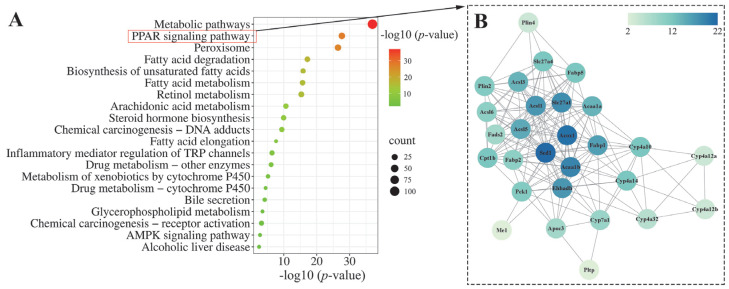
Bioinformatics analysis of the DEPs between the HPG and DCG. (**A**) KEGG pathway analysis of the DEPs and (**B**) PPI network analysis of the DEPs enriched in the PPAR signaling pathway.

To further elucidate the potential mechanism of action of LLPK in ameliorating hepatic lipid accumulation in *db*/*db* mice, we performed a KEGG pathway analysis on DEPs between HPG and DCG. The 20 most significantly enriched KEGG pathways among the DEPs between HPG and DCG are presented in Figure 5A. It was noteworthy that a significant number of DEPs were involved in pathways associated with metabolic processes. Notably, fatty acid degradation and fatty acid metabolism were predominant, particularly highlighting the PPAR signaling pathway. Consequently, we performed a protein–protein interaction (PPI) network analysis on these 28 DEPs enriched in the PPAR signaling pathway using the online analytical tool String. Finally, we obtained a PPI network graph containing 28 nodes and 161 degrees, as illustrated in Figure 5B.

**Figure 6:**
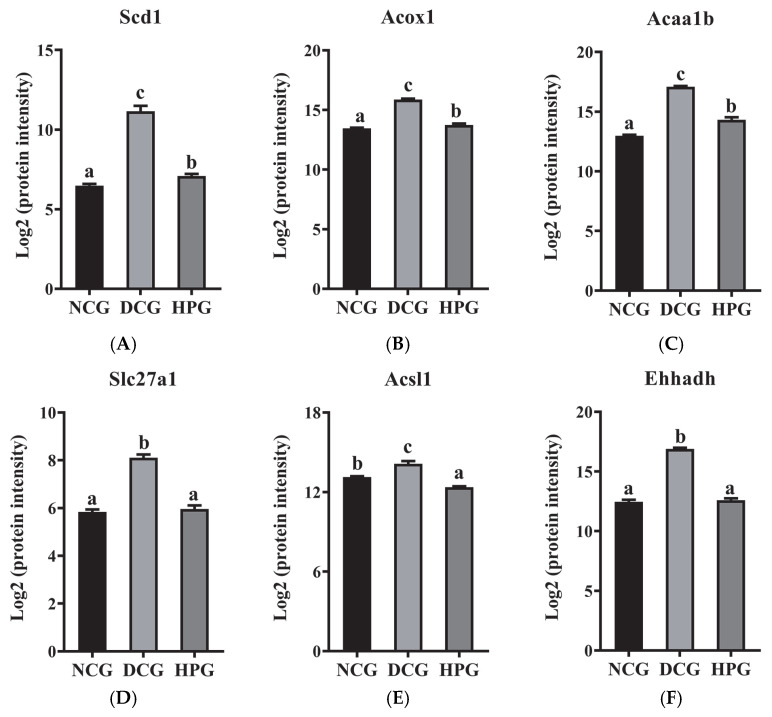
The log_2_ protein intensity of (**A**) Scd1, (**B**) Acox1, (**C**) Acaa1b, (**D**) Slc27a1, (**E**) Acsl1, and (**F**) Ehhadh from the hepatic proteome (the letter superscripts indicate the significant differences among samples based on ANOVA analysis, *p* < 0.05).

Of these, acyl-CoA desaturase 1 (Scd1), acyl-coenzyme A oxidase 1 (Acox1), 3-ketoacyl-CoA thiolase B, peroxisomal (Acaa1b), long-chain fatty acid transport protein 1 (Slc27a1), long-chain-fatty-acid-CoA ligase 1 (Acsl1), and peroxisomal bifunctional enzyme (Ehhadh) were the nodes that exhibited the highest degree of interaction with other proteins. Subsequently, these six DEPs were quantified based on the mass spectral intensities provided by proteomics (Figure 6). Among these six identified proteins, Scd1 is associated with lipogenesis. The levels of hepatic Scd1 in DCG were significantly overexpressed compared with NCG (Figure 6B, *p* < 0.05). Notably, this phenomenon was suppressed in the mice given high doses of LLPK, where the expression level of hepatic Scd1 was significantly decreased in HPG mice compared with DCG (*p* < 0.05). Furthermore, we quantified Acox1, Acaa1b, and Ehhadh, which are crucial proteins involved in fatty acid oxidation. As illustrated in Figure 6B, the expression levels of Acox1, Acaa1b, and Ehhadh were markedly elevated in the livers of DCG in comparison to NCG (*p* < 0.05). Following a 30-day period of LLPK intervention, a significant suppression in the expression levels of hepatic Acox1, Acaa1b, and Ehhadh was observed in HPG (*p* < 0.05). Similarly, a comparable phenomenon was observed in Slc27a1 and Acsl1, where the elevated expression levels of Slc27a1 and Acsl1 in DCG were significantly suppressed (*p* < 0.05) in HPG.

## 4. Discussion

Camel milk protein has been shown to be a good source of peptides with antihypertensive and anti-diabetic bioactivities [14]. Our previous research has demonstrated the hypoglycemic effect of camel milk in HFD/STZ-induced diabetic rats [7]. Based on this finding, a following study from our group has identified an active peptide LLPK from camel milk protein, exerting excellent DPP-4 inhibitory activity in vitro. However, the hypoglycemic effect of LLPK in vivo is not clear. Therefore, we synthesized the highly-pure active peptide LLPK and used *db*/*db* diabetic mice to evaluate its in vivo anti-diabetic activity.

As a result, the *db*/*db* mice utilized in this study exhibited typical manifestations of diabetes, including hyperglycemia, hyperlipidemia, and IR after 30 consecutive days of feeding compared to normal control mice, which is similar with a previous finding [15]. However, LLPK treatment significantly suppressed FBG levels in *db*/*db* mice. Additionally, the results of OGTT, ITT, HOMA-IR, and HOMA-IS confirmed the potent ameliorative effects of high-dose LLPK treatment on improving insulin sensitivity and reducing IR in diabetic mice (Table 1, Figure 2). These beneficial effects may contribute to the inhibition of DPP-4 activity and the elevation of serum GLP-1 levels in *db*/*db* mice [16,17]. Similar findings were observed by Rai et al. [18], who reported that whey protein hydrolysate treatment could modulate GLP-1 levels by inhibiting DPP-4 expression, thereby ameliorating hyperglycemia and IR in obese mice. Furthermore, GLP-1 is an enteric glucagon that delays gastric emptying and suppresses appetite via the central nervous system [17]. We observed a significant reduction in food intake in *db*/*db* mice after gavage of LLPK, which corresponded to significantly lower body weights in both LPG and HPG compared to DCG.

In the present study, low-dose and high-dose LLPK treatments both effectively reduced lipid levels in *db*/*db* mice (Table 1), suggesting that LLPK possessed anti-hyperlipidemic activity. T2DM frequently occurs with disorders of lipid metabolism [19]. These abnormalities have also been observed in cases of IR [20]. Moreover, significant lipid droplet deposition was observed in the liver of DCG mice, with hepatocytes displaying signs of edema and vacuolation (Figure 3C). Following 30 days of LLPK treatment, both LPG and HPG resulted in a significant liver weight loss when compared to DCG (Figure 3A). It is particularly susceptible to alterations in glucose-lipid metabolism during the pathogenesis of diabetes, leading to the accumulation of lipid metabolic products and the development of aberrant lesions in liver tissue [7,21]. Additionally, the signs of edema and vacuolation in the hepatocytes of *db*/*db* diabetic mice were notably diminished in both LPG and HPG (Figure 3E,F). Similar findings were reported by Zhang et al. [8], who demonstrated that camel milk polypeptide treatment ameliorated lipid metabolism disorders by modulating intestinal flora composition in HFD/STZ-induced diabetic mice, thereby reducing hepatic lipid accumulation. These results demonstrated that LLPK treatment played a beneficial role in regulating disorders of glycolipid metabolism in *db*/*db* diabetic mice.

To gain insight into the mechanism by which LLPK treatment improves glycolipid metabolism regulation, we conducted a proteomic analysis of liver tissue. Our results indicated that the DEPs between HPG and DCG were primarily associated with the PPAR signaling pathway, fatty acid metabolism, and other glycolipid metabolism pathways. The PPAR signaling pathway plays a critical role in controlling lipid synthesis, catabolism, and efflux. Thus, dysregulation of the PPAR signaling pathway may result in dyslipidemia, diabetes mellitus, etc. [22]. PPARα, PPARδ, and PPARγ are three isoforms in the PPAR family. Of them, PPARα is predominantly expressed in the liver, which is essential for regulating glycolipid metabolism [22]. Although the PPARα expression was detected in the livers of *db*/*db* mice using proteomic methods in this work, there was no marked difference in the expression levels of hepatic PPARα between different groups (Table S1). Therefore, we speculated that LLPK may not directly regulate PPARα expression to mediate the PPAR signaling pathway. To investigate how LLPK mediates the PPAR signaling pathway, we employed PPI network analysis. The results presented in Figure 5B indicated that Scd1, Acox1, Acaa1b, Slc27a1, Acsl1, and Ehhadh were the DEPs with the highest degree of interaction. As these proteins are the downstream targets regulated by PPARα, LLPK may modulate PPAR signaling pathway through the regulation of these downstream proteins, rather than directly affecting the expression of PPARα.

Overexpression of Scd1 could induce metabolic issues such as IR and T2DM [23]. The present work revealed that Scd1 expression levels were markedly elevated in *db*/*db* mice, which were consistent with previous findings [24,25]. Notably, *db*/*db* mice treated with LLPK exhibited a significant decline in the hepatic Scd1 level. A prior investigation by Görgens et al. demonstrated that targeted silencing of hepatic DPP-4 expression led to a substantial reduction in hepatic Scd1 mRNA levels [24]. It was therefore hypothesized that LLPK may decrease hepatic Scd1 expression by inhibiting the expression or activity of hepatic DPP-4, thereby ameliorating hepatic lipid accumulation. On the other hand, Acox1, Acaa1b, and Ehhadh are key proteins involved in fatty acid oxidation [26,27,28]. A recent report indicated that a high-fat diet could induce elevated mRNA levels of Acox1 in mouse liver [26]. Meanwhile, specific knockdown of hepatic Acox1 expression prevented obesity, IR, and related complications in mice with HFD [26]. He et al. [29] reported a similar finding, who demonstrated that a high-fat diet increased hepatic Acox1 gene expression. However, hepatic Acox1-LKO was shown to induce fat autophagy, thereby preventing fatty liver [29]. Although studies on the mechanisms by which active peptides regulate Acaa1b and Ehhadh and thereby affect lipid metabolism are scarce, some research regarding the bioactivity of other active compounds have been published. A recent study demonstrated that dark tea extract could ameliorate glycolipid metabolism disorders in *db*/*db* mice by mediating the expression of key factors in the PPAR signaling pathway, such as Ehhadh and Fabp1 [30]. Similarly, this study revealed a notable reduction in hepatic Acox1, Acaa1b, and Ehhadh expression levels of *db*/*db* mice following LLPK treatment (Figure 6). It can therefore be postulated that the regulation of fatty acid oxidation may serve as a key mechanism through which LLPK exerts its beneficial effects on lipid metabolism disorders.

Moreover, one interesting thing to note is that the expression levels of hepatic Slc27a1 and Acsl1 were found to be notably reduced in *db*/*db* mice following LLPK treatment (Figure 6). Some previous studies have confirmed that Slc27a1 and Acsl1 serve as major regulatory factors, playing an indispensable role in the regulation of hepatic lipid metabolism by mediating the transport of fatty acids [31,32]. Furthermore, Pfohl et al. demonstrated that treatment of high-fat diet mice with a phenolic-rich pomegranate extract significantly suppressed the expression of Fabp1, Slc27a1, and Cd36 (which are involved in fatty acid transport), thereby limiting the accumulation of fatty acids in the mice livers [33]. Another report demonstrated that the preventive effect of Evogliptin (a DPP-4 inhibitor) on cardiac lipotoxicity in *db*/*db* mice may be closely associated with the inhibition of genes such as ACSL1, FABP3, and PPARγ, which in turn reduced the accumulation of lipid droplets in the myocardium [15]. Conversely, the aberrant lipid metabolism has been associated with the overexpression of Slc27a1 and Acsl1 in HFD-induced obese mouse models [33] and *db*/*db* mouse models [15]. These results all demonstrated that fatty acid transport mediated by Slc27a1 and Acsl1 may play an integral role in the beneficial effects on lipid metabolism disorders by LLPK.

## 5. Conclusions

**Figure 7:**
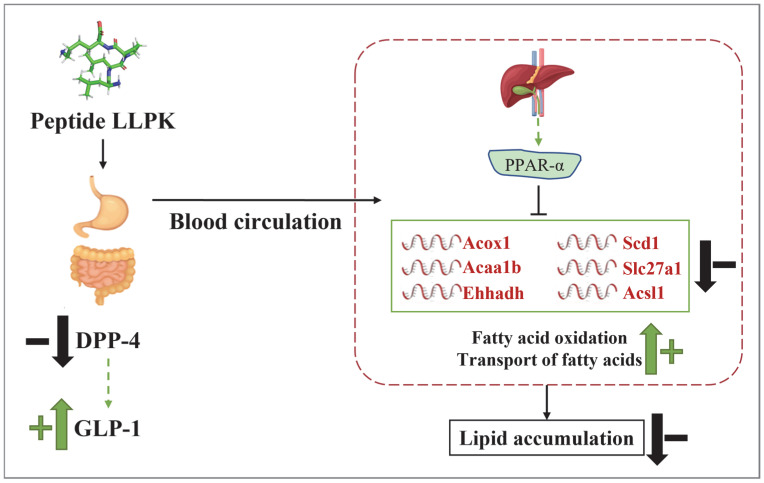
The possible mechanism by which LLPK ameliorates lipid accumulation through the PPAR signaling pathway.

The present study demonstrated that the camel milk protein-derived peptide LLPK enhances insulin sensitivity and regulates glucolipid metabolic homeostasis in *db*/*db* mice by inhibiting DPP-4 activity and elevating serum GLP-1 levels. Hepatic proteomics reveal that LLPK modulates hepatic lipid metabolism by mediating the PPAR signaling pathway, thereby reducing hepatic lipid accumulation. Specifically, LLPK intervenes in biological processes such as lipogenesis, fatty acid oxidation, and fatty acid transport in mice livers by regulating the expression of key downstream factors of PPARα (Scd1, Acox1, Acaa1b, Slc27a1, Acsl1, and Ehhadh), thus improving lipid metabolic disorders (Figure 7). These findings provide a new insight for the use of camel milk protein-derived peptides as potential anti-diabetic compounds, and support the development of camel milk-based functional foods or nutraceuticals.

## Figures and Tables

**Table 1:** Effect of LLPK treatment on plasma biochemical parameters in *db*/*db* diabetic mice.

	NCG	DCG	PCG	LPG	HPG
TC (mmol/L)	6.72 ± 0.16 ^a^	15.63 ± 0.81 ^d^	7.63 ± 0.57 ^b^	9.77 ± 1.00 ^c^	10.53 ± 1.09 ^c^
TG (mmol/L)	5.14 ± 0.39 ^b^	7.5 ± 0.36 ^d^	5.51 ± 0.22 ^c^	5.44 ± 0.3 ^bc^	4.79 ± 0.19 ^a^
HDL-C (mmol/L)	3.17 ± 0.45 ^c^	1.44 ± 0.32 ^a^	2.82 ± 0.91 ^c^	1.78 ± 0.68 ^b^	1.26 ± 0.53 ^b^
LDL-C (mmol/L)	3.40 ± 0.43 ^a^	12.89 ± 0.65 ^d^	4.96 ± 0.79 ^b^	7.53 ± 1.06 ^c^	7.72 ± 1.13 ^c^
Insulin (mIU/L)	8.35 ± 0.24 ^c^	9.96 ± 0.29 ^d^	7.63 ± 0.47 ^ab^	7.75 ± 0.45 ^ab^	7.93 ± 0.23 ^b^
HOMA-IR	2.44 ± 0.27 ^a^	11.64 ± 0.48 ^d^	3.53 ± 0.77 ^b^	6.5 ± 1.39 ^c^	4.29 ± 1.38 ^b^
HOMA-IS	0.0192 ± 0.0016 ^d^	0.0038 ± 0.0001 ^a^	0.0142 ± 0.0022 ^c^	0.0085 ± 0.0010 ^b^	0.0112 ± 0.0031 ^bc^
GLP-1 (pmol/L)	0.87 ± 0.04 ^bc^	0.80 ± 0.03 ^a^	1.10 ± 0.07 ^d^	0.83 ± 0.05 ^ab^	0.90 ± 0.06 ^c^
DPP-4 activity (mU/mL)	5.72 ± 0.46 ^a^	29.95 ± 0.93 ^d^	6.45 ± 0.35 ^a^	23.33 ± 1.82 ^c^	13.51 ± 0.33 ^b^

Data presented as means ± SD (n = 6); values with different superscripts represent significant differences (*p* < 0.05).

## Data Availability

The original contributions presented in this study are included in the article/Supplementary Material. Further inquiries can be directed to the corresponding author.

## Supplementary Materials

The following supporting information can be downloaded at: https://www.mdpi.com/article/10.3390/nu17101693/s1, Table S1: In vitro DPP-4 activity inhibitory capacity of peptides identified in high-activity fractions of camel milk hydrolysate. Table S2. The list of all identified proteins from hepatic proteome.

## Abbreviations

The following abbreviations are used in this manuscript:

Acox1	acyl-coenzyme A oxidase 1
Acaa1b	3-ketoacyl-CoA thiolase B
Acsl1	long-chain-fatty-acid-CoA ligase 1
DPP-4	dipeptidyl-peptidase 4
DCG	diabetic control group
DEP	different expressed proteins
Ehhadh	peroxisomal bifunctional enzyme
FBG	fasting blood glucose
GLP-1	glucagon-like peptide 1
HPG	high-dose peptide treatment group
ITT	insulin tolerance test
LLPK	Leucine-Leucine-Proline-Lysine
LPG	low-dose peptide treatment group
NCG	normal control group
OGTT	oral glucose tolerance tests
PCG	metformin control group
PCA	principal component analysis
PPI	protein–protein Interaction
Scd1	acyl-CoA desaturase 1
Slc27a1	long-chain fatty acid transport protein 1
T2DM	type 2 diabetes
