# Supplementary material for: A Novel Camel Milk-Derived Peptide LLPK Improves Glucose-Lipid Metabolism in db/db Mice via PPAR Signaling Pathway
Source: Nutrients. 2025 May 16;17(10):1693. doi: 10.3390/nu17101693 (PMC12113985; doi:10.3390/nu17101693)
Supplement: Supplementary file 1 [file nutrients-17-01693-s001.zip › Tabel S1.pdf]

Table 1. *In vitro* DPP4 activity inhibitory capacity of peptides identified in high activity fractions of camel milk hydrolysate

No.	Peptide	Protein precursor	Mass (Da)	error (ppm)	Area	DPP4 inhibitory IC_{50} (mM)
1	HKPL	α-La	493.3012	-0.5	1.61E+07	>200
2	AHKPL	α-La	564.3384	-0.8	2.30E+07	9.19±0.19[c]
3	AVPK	XDH	413.2638	-0.2	3.78E+06	>200
4	RTF	XDH	422.2278	-0.9	1.29E+06	0.15±0.02[b]
5	LLPK	ALB	469.3264	1.2	1.52E+08	0.11±0.01[a]
6	LKPEPE	ALB	711.3802	-0.4	7.18E+06	>200
7	HPVPQP	β-CN	673.3547	-1.3	1.45E+07	>200
8	LKPEPEA	ALB	782.4174	-1.6	1.32E+07	>200
9	YDTPEG	α-S1-CN	680.2653	-2	4.62E+06	>200
10	YYPPQ	α-S1-CN	666.3013	-2.4	8.51E+08	30.01±3.05[d]
11	MIPPQS	β-CN	671.3312	-3.2	1.42E+08	>200

α-La: α-lactalbumin, XDH: xanthine dehydrogenase, ALB: albumin, β-CN: β-Casein, α-S1-CN: α-S1-Casein

DPP4 inhibition IC_{50}, concentration resulting in 50% DPP-IV inhibition (mM). Different letters (a–d) represent significant differences at $p < 0.05$.
